# Safety and feasibility of the treatment of calcified *de novo* coronary artery lesions with drug-coated balloon angioplasty after intravascular lithotripsy

**DOI:** 10.3389/fcvm.2026.1753826

**Published:** 2026-01-30

**Authors:** Alma Räsänen, Antti Eranti, Tuomas T. Rissanen

**Affiliations:** 1 Heart Center, North Karelia Central Hospital, Joensuu, Finland; 2 University of Eastern Finland, Joensuu, Finland

**Keywords:** bleeding risk, calcified lesions, drug-coated balloon, drug-eluting balloon, lithotripsy, myocardial infarction

## Abstract

**Objectives:**

Percutaneous coronary intervention (PCI) of calcified lesions using stenting may lead to stent malapposition and stent underexpansion. The combination of intravascular lithotripsy (IVL) followed by drug-coated balloon (DCB) treatment may help overcome this limitation. The aim of this single-center, retrospective, registry-based observational study was to assess the efficacy and safety of plaque modification using IVL followed by DCB-only treatment in patients with severely calcified lesions.

**Methods:**

Severely calcified *de novo* coronary artery lesions were prepared using IVL followed by the application of paclitaxel-coated DCB in 34 consecutive patients; five patients requiring bail out stenting were excluded from the analysis. The cohort included patients both with stable coronary artery disease (53%) and acute coronary syndromes (47%). The mean age of the patients was 75 years and 56% had diabetes. The majority of patients (76%) were at high bleeding risk based on the Academic Research Consortium criteria. The primary endpoint was MACE [major adverse cardiac events, defined as a composite of target lesion revascularization (TLR), myocardial infarction (MI), and cardiovascular (CV) mortality] at 12 months. The secondary endpoints included individual components of MACE at 6 and 12 months and ARC bleeding (BARC) events.

**Results:**

There were no acute vessel closures or perioperative myocardial infarctions. During 12-month follow-up, the primary end point occurred in 15% (*n* = 5) of the patients, primarily driven by CV death (9%, *n* = 3) and one type-2 MI (3%). There was only one ischemia driven TLR within 12 months (3%). The rate of Bleeding Academic Research Consortium (BARC) 2–5 and BARC 3–5 bleeding events was 24% and 6% at twelve months, respectively.

**Conclusions:**

PCI using IVL in combination with an application of paclitaxel-DCB strategy was feasible in the treatment of severely calcified coronary artery lesions in this cohort. This novel approach may be particularly advantageous for patents at high risk of bleeding, although further studies are needed to confirm this potential benefit.

## 1 Introduction

Obstructive coronary artery disease (CAD) with heavily calcified lesions presents a significant challenge for percutaneous coronary intervention (PCI). As PCI is increasingly performed on elderly patients, the prevalence of severe coronary calcification continues to rise, particularly among individuals with comorbidities such as diabetes and renal insufficiency (1). Calcified lesions increase the complexity and risks associated with PCI due to the rigidity and inflexibility of calcified plaque, which compromises standard procedural approaches (2). The long-term adverse outcomes also increase with the severity of coronary calcification (3).

For severely calcified coronary lesions, standard balloon angioplasty techniques, such as semi-compliant (SC) and non-compliant (NC) balloon inflation, are frequently insufficient. In these cases, additional devices, including rotational atherectomy (RA), orbital atherectomy (OA), or intravascular lithotripsy (IVL), are often required (2, 4). RA and OA involve ablation of the calcified plaque but require specialized training and expertise, and may result in complications such as no-reflow (5). In contrast, intravascular lithotripsy, a newer modality, offers an accessible, *ad hoc* solution that can be employed when standard predilatation balloons are ineffective (6).

IVL is a relatively new method designed to treat calcified coronary lesions, addressing certain limitations of traditional balloon angioplasty. IVL catheters contain emitters that produce pulsating shockwaves. These shockwaves are created by an electrical discharge that vaporizes the fluid inside the balloon, forming rapidly expanding and collapsing bubbles. The mechanical energy generated is transferred to the vessel wall, effectively breaking up calcifications even in the deeper layers. Compared to atherectomy techniques, IVL is associated with fewer complications, such as guidewire bias, and often provides more consistent lesion preparation with a lower risk of periprocedural complications (7). Thus far, IVL has been mainly studied in association with the implantation of drug-eluting stents (DES).

## 2 Methods

### 2.1 Study design and population

**Figure 1:**
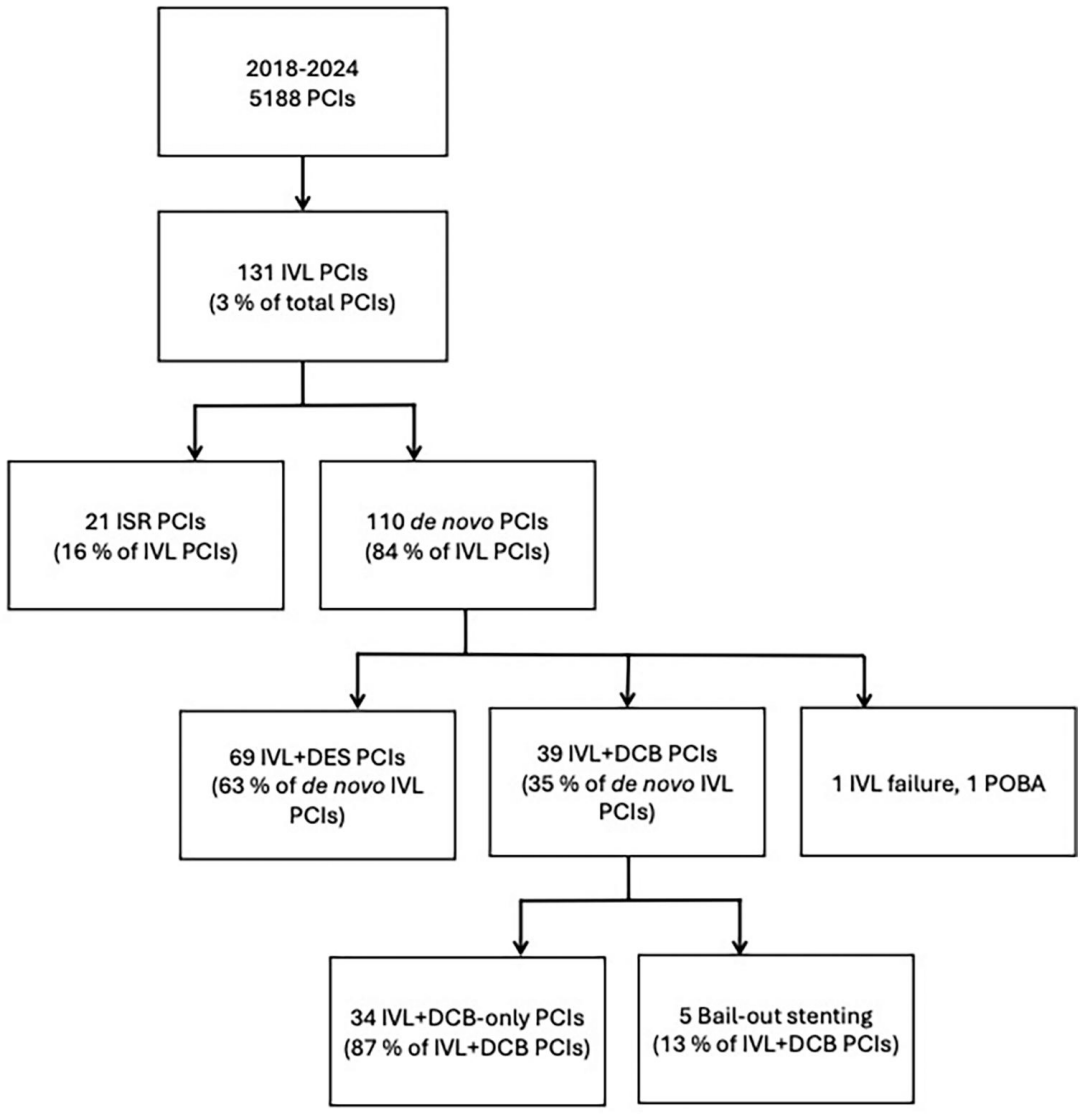
Flow chart of the study. PCI, percutaneous coronary intervention; IVL, intravascular lithotripsy; ISR, in-stent restenosis; DES, drug-eluting stent; DCB, drug-coated balloon; POBA, plain old balloon angioplasty.

A total of 39 PCIs in 34 patients were conducted on severely calcified lesions using intravascular lithotripsy followed by paclitaxel DCB in our institution. All consecutive patients treated between July 2018 and August 2024 were included in the analysis. The flowchart of the study is shown in the Figure 1. The exclusion criterion for this study was the need for a DES implantation after lesion predilatation, such as a flow limiting dissection (TIMI < 3) or a significant recoil (>30%) (8). Lesion location was not an exclusion criterion, and all treated lesions were *de novo*. The study was approved by the institutional review board.

### 2.2 PCI using IVL followed by DCB

The use of IVL was at the operator's discretion: either upfront due to severe calcification as evaluated angiographically or by intravascular imaging, or as a bailout strategy due to an undilatable lesion. IVL was performed using the Shockwave™ balloon catheter (Shockwave, USA, California). The IVL procedure was conducted in accordance with the manufacturer's instructions. The inflation pressure of the IVL balloon was 4 ATM. If the IVL balloon appeared undersized, the inflation pressure was increased to achieve angiographical 1:1 sizing. After IVL, the lesion was further dilated using NC- or cutting balloon with the balloon-reference size of 1:1. If the lesion was uncrossable for the Shockwave balloon, a small SC-balloon or rotational atherectomy (15%) was used before IVL at the operator's discretion. The lesions with an acceptable predilatation result (less than 30% recoil or flow-limiting dissection) were then treated with DCB, which was conducted in accordance with the international consensus documents (8, 9). DCB inflation time was at least 30 s. The DCBs used in this study were paclitaxel-coated. In 13% of patients, two lesions were treated with IVL and DCB. The left anterior descending artery (LAD) was the most common target vessel (67%).

## 3 Endpoints

Postoperative patient care and follow-up were conducted in accordance with standard local practices. The primary endpoint of this study was MACE [major adverse cardiac events: composite of target lesion revascularization (TLR), cardiovascular (CV) mortality and myocardial infarction (MI)] at 12 months. The secondary endpoints were the individual components of MACE at 6 and 12 months. Also, total mortality, acute vessel thrombosis, target vessel revascularization, incidence of stroke and Bleeding Academic Research Consortium (BARC) 2–5 and BARC 3–5 bleedings were studied. Myocardial infarction was classified according to the fourth universal definition of MI (10). Bleeding events were categorized following the Bleeding Academic Research Consortium (BARC) criteria (11).

Our center serves a population of approximately 170.000 inhabitants, with all regional patients requiring coronary angiography referred to our institution. The central hospital and all regional health centers use a unified electronic medical record system, providing comprehensive access to patient data and laboratory parameters throughout follow-up. Data regarding the cause of death were also retrieved from the medical records, and all causes of death were documented. No patients were lost on the follow-up. The clinical endpoints were adjudicated by the research group.

Categorical variables were presented as counts and percentages [*n* (%)], while continuous variables were expressed as means with standard deviation or as medians. Cumulative MACE rates and BARC 2–5 bleeding events were analyzed using the Kaplan–Meier method.

## 4 Results

### 4.1 Study population

The patients presented with either stable coronary artery disease (CAD, 53%) or acute coronary syndrome (ACS, 47%), including unstable angina (31%), non-ST-elevation myocardial infarction (63%) or ST-elevation myocardial infarction (6%) (Table 1). The patients were elderly with the mean age 75 years and frequently had comorbidities such as diabetes (56%), renal insufficiency (79%), and 44% of the patients had a reduced ejection fraction. Moreover, the majority (76%) of patients were at a high bleeding risk (HBR) according to the Bleeding Academic Research Consortium criteria (BARC). At the time of the PCI, 38% of patients were on oral anticoagulation (OAC).

**Table 1:** Baseline characteristics of the patients.

Patient charasteristics	*n* or mean	%
Number of patients	34	
Age, mean ± SD, years	75 ± 7	
Sex, male	25	74
Risk factors for coronary artery disease		
Current smoker	3	9
Ex-smoker	10	29
Diabetes	19	56
Hypertension	29	85
Hypercholesterolemia (LDL > 2,5 mmol/L or on statin)	32	94
Stable coronary artery disease	18	53
Acute coronary syndromes (NSTEMI, UAP, STEMI)	16	47
STEMI	1	6
NSTEMI	10	63
UAP	5	31
High bleeding risk (ARC)	26	76
OAC	13	38
Hemoglobin, mean ± SD g/L	126 ± 24	
Ejection fraction^b^		
≥55%	18	55
30–54%	11	33
<30%	4	12
Glomerular filtration rate (mL/min/1.732 m^2^)^a^		
60–90	14	52
30–59	11	41
<30	2	7
Follow-up angiography^c^	12	35

All data presented as *n* (%). OAC, oral anticoagulant; ARC, the Academic Research Consortium criteria.

a Chronic kidney Disease Epidemiology Collaboration (CKD-EPI) formula.

b Data available on 33 patients.

c Follow-up angiography images available on 11 patients, one follow-up CAG only text based.

### 4.2 Lesion characteristics

The lesion and procedural characteristics are shown in Table 2. The treated vessels were large as the mean IVL diameter was 3.2 mm (±0.3 SD), the mean diameter of the predilation balloon was 3.2 mm (±0.4 SD), and the mean diameter of DCB was 3.3 mm (±0.4 SD). Total of 52 DCBs were used (SeQuent Please Neo, B. Braun, Germany; *n* = 50, 96% or Pantera Lux, Biotronik, Germany; *n* = 2, 4%). Intravascular imaging (OCT, Optical Coherence Tomography or IVUS, Intravascular ultrasound) was used in 6 PCIs (18%). Rotational atherectomy prior to IVL treatment was used in 5 PCIs (15%) and cutting balloon in 15 lesions (38%). Six of the lesions were classified as true bifurcation lesions (Medina classification 1.1.1, 1.0.1, or 0.1.1) (12). The lesions were predominantly complex, as 79% were categorized as type B2 or C. Additionally, 8% of the lesions were chronic total occlusions (CTO). Almost all lesions (97%) were classified as severely calcified involving calcification on both sides of the arterial lumen according to the Mintz classification (13).

**Table 2:** Lesion and procedural characteristics.

Lesion and procedural characteristics	*n* or mean	%
Number of patients	34	
Total number of lesions treated with PCI	39	
Total number of DCBs used	52	
2 or more lesions	5	15
Target vessel		
LAD	26	67
LCX	6	15
RCA	5	13
Diagonal	1	3
Marginal	1	3
Severe calcification^b^	33	97
Cutting balloon	15	38
Rotational atherectomy	5	15
Intravascular imaging		
OCT	3	9
IVUS	3	9
True bifurcation lesion^a^	6	15
ACC/AHA classification		
B1	5	13
B2	15	38
C	16	41
CTO	3	8
Balloon size ± SD mm		
IVL balloon diameter	3.2 ± 0.3	
NC balloon diameter	3.2 ± 0.4	
DCB diameter	3.3 ± 0.4	
SeQuent Please Neo DCB	50	96
Pantera Lux DCB	2	4
Post-PCI antiplatelet treatment		
Triple therapy		
DAPT + OAC	1	3
Double therapy^c^	29	85
DAPT	18	53
SAPT + OAC	11	32
Single therapy	4	12
SAPT alone	2	6
OAC alone	2	6
Mean double therapy duration (months)^c^	5 ± 5	
Mean double therapy duration on CCS patients	8 ± 5	
Mean double therapy duration on ACS patients	3 ± 3	

PCI, percutaneous coronary intervention; DCB, drug coated balloon; LAD, left anterior descending artery; LCX, left circumflex artery; RCA, right coronary artery; OCT, optical coherence tomography; IVUS, intravascular ultrasound; IVL, intravascular lithotripsy; NC, non-compliant balloon; DCB, drug coated balloon; DAPT, dual antiplatelet treatment; OAC, oral anticoagulation; SAPT, single antiplatelet treatment; CCS, chronic coronary syndrome; ACS, acute coronary syndrome.

a Defined as Medina 1.1.1, 1.0.1, or 0.1.1.

b Minz classification.

c Double therapy consists of DAPT and SAPT + OAC.

### 4.3 Survival and MACE rates after PCI

There were no incidents of acute vessel closure or perioperative myocardial infarction. At the 12-month follow-up, the primary endpoint—major adverse cardiovascular events (MACE)—occurred in 15% of patients (*n* = 5), primarily driven by cardiovascular death (9%, *n* = 3). The incidence of MACE was significantly higher (25%, *n* = 4) among patients presenting with acute coronary syndrome (ACS) as compared to patients with chronic coronary syndrome (CCS, 6%, *n* = 1). Within 12 months, one myocardial infarction (MI) occurred, as well as one ischemia-driven target lesion revascularization (TLR). Moreover, one LAD CTO lesion treated with IVL + DCB were found to be reoccluded one year two months after index PCI, respectively. The only myocardial infarction within 12 months in this population was classified as a type 2 infarction, and the index lesion was stable with no need for reintervention. The MACE rate at 6 months was 9% (*n* = 3).

Overall mortality was 18% at twelve months (*n* = 6), attributable to the advanced age and comorbid conditions of the study population.

### 4.4 Antiplatelet therapy and bleeding after PCI

A total of 53% (*n* = 18) of patients were discharged on dual antiplatelet therapy (DAPT), 3% (*n* = 1) on triple therapy (DAPT and oral anticoagulation, OAC), and 32% (*n* = 11) on single antiplatelet therapy (SAPT) combined with permanent OAC. 6% (*n* = 2) was discharged on SAPT and 6% (*n* = 2) on OAC only. Mean duration of double therapy (DAPT or SAPT + OAC) was 5 ± 5 months in all patients, and 3 ± 3 months and 8 ± 5 months among in CCS patients and among ACS patients, respectively. 12% of patients (*n* = 4) were discharged with permanent dual therapy (DAPT or OAC + SAPT) (Table 2). All patients discharged on SAPT were at HBR. All the MACE events were on patients discharged on dual therapy.

**Figure 2:**
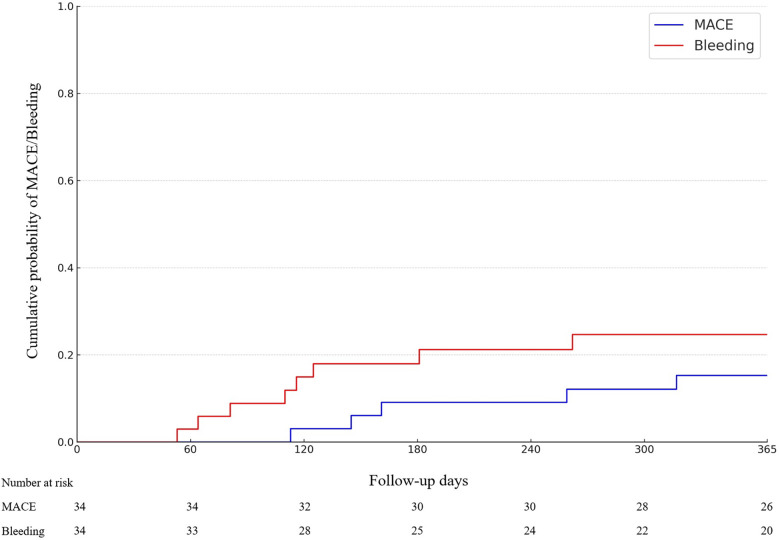
Cumulative incidence of Major adverse cardiac events (MACE) and BARC2-5 bleeding-events (*p* = NS).

**Figure 3:**
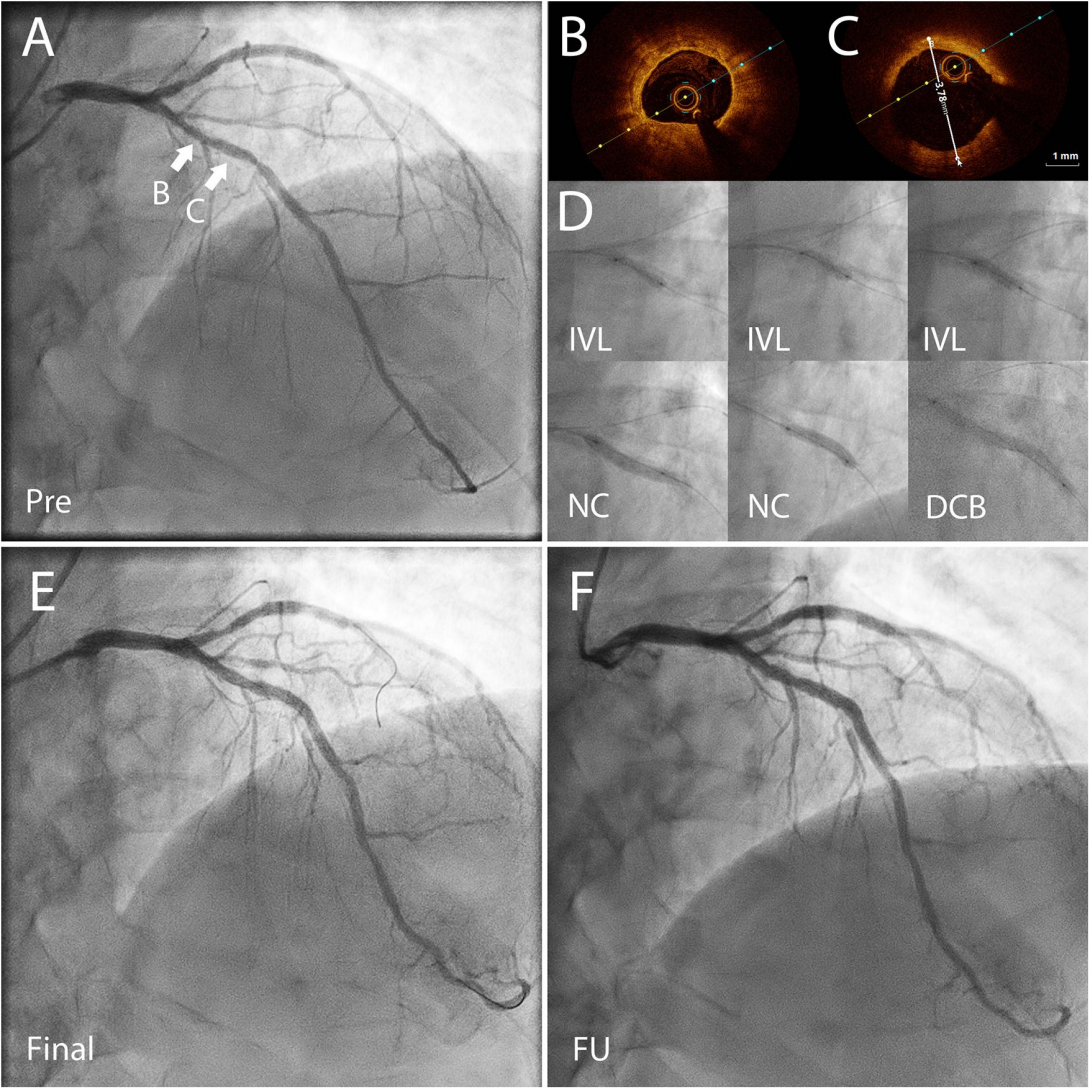
Coronary angiography (CAG) and optical coherence tomography (OCT) images of a 72 yr old patient presenting with CCS3 chest pain. The risk factors for coronary artery disease included high blood pressure and hypercholesterolemia. He had a high bleeding risk due to a permanent anticoagulation with warfarin treatment (APC resistance). **(A)** CAG before percutaneous coronary intervention (PCI) shows severe coronary stenosis in the left anterior descending (LAD) artery. FFR was 0.72 in the distal LAD demonstrating significant reversible myocardial ischemia. **(B)** OCT before PCI showed concentric calcification. **(C)** OCT before PCI showed the vessel reference diameter of 3.78 mm distally from the lesion B. **(D)** First, non-compliant (NC) and OPN NC balloons were used but they bursted. Thereafter, the severely calcified lesion was predilated using with 3.5 × 12 mm Shockwave™ balloon for 80 pulses, and then further predilation was performed with a 3.5 mm NC-balloon. After predilation there was no significant recoil (>30%) or flow-limiting dissection, and the lesion was treated with two paclitaxel drug-coated balloons (DCB) 3.5 × 40 mm and 3.5 × 15 mm for 60 s. **(E)** The final result after IVL + DCB treatment. **(F)** CAG almost two years after the index procedure done before planned aortic valve replacement surgery demonstrating a good long-term result of the treated area. CCS, Canadian cardiovascular society angina grade; PCI, percutaneous coronary intervention; FFR, fractional flow reserve; OCT, optical coherence tomography; OPN NC, super high pressure non-compliant balloon catheter; IVL, intravascular lithotripsy.

Clinically significant bleeding events (BARC 2–5) occurred in 24% of the patients (*n* = 8), and severe bleeding events (BARC 3–5) in 6% (*n* = 2) of the patients. Most common bleeding events were gastrointestinal bleedings. 88% of the BARC 2–5 bleeding events occurred on patients in dual therapy. The primary and secondary endpoints of the study are shown in Table 3 and demonstrated in Figure 2. One case example of IVL followed by DCB-only PCIs is described in Figure 3. Pre, post IVL + DCB treatment and follow-up coronary angiographies are shown in the Supplementary Figure S1.

**Table 3:** The primary and secondary endpoints of the study at 6 and 12 months.

End points	6 months		12 months	%
	*n*	%	*n*	
MACE	3	9	5	15
Cardiovascular mortality	2	6	3	9
Myocardial infarction	1	3	1	3
TLR	0	0	1	3
TVR	0	0	0	0
Stroke	1	3	1	3
BARC 2–5 bleeding	7	21	8	24
BARC 3–5 bleeding	2	6	2	6
Total mortality	3	9	6	18

MACE, major adverse cardiovascular event comprising of cardiovascular death, non-fatal myocardial infarction, and target lesion revascularization (TLR); TVR, Target vessel revascularization; BARC, bleeding academic research consortium.

## 5 Discussion

Calcified coronary lesions remain a significant challenge of PCI, often leading to poorer outcomes compared to non-calcified lesions (1). These challenges are largely due to difficulties in lesion preparation, such as the inability to effectively cross and dilate the lesion using standard devices (e. g., semi-compliant or non-compliant balloon). The rigidity of calcified lesions also increases the risk of complications, including vessel perforation, especially when using aggressive pre-dilation or atherectomy techniques (1, 2, 14, 15). Additionally, calcified plaques can hinder stent expansion and positioning, increasing the risk of stent malapposition. This, in turn, causes the risk of stent thrombosis and restenosis, both of which are associated with adverse clinical outcomes (16–19). Achieving optimal stent deployment in these cases typically requires specialized adjunctive tools designed to effectively treat calcified lesions. DCB technologies have been developed more recently for non-stent based local drug delivery of antiproliferative drugs in order to reduce the risk of neointimal growth and restenosis (20, 21). The potential advantages of DCB-only angioplasty include more uniform antiproliferative drug delivery to the vessel wall, the possibility of later positive remodeling, preservation of coronary vasomotion and the absence of risk for stent malapposition or thrombosis. So far, the data with IVL combined with DCB -angioplasty has been limited and only with short follow up (22).

In the present study, we investigated the clinical outcomes of consecutive patients with calcified lesions treated with IVL followed by paclitaxel-DCB. The results suggest that this novel approach is feasible and safe for treating calcified complex coronary stenoses. Paclitaxel is a lipophilic drug that rapidly crosses the cell membrane and binds to microtubules, thus inhibiting cell division, migration and proliferation. Its lipophilic properties ensure rapid cellular uptake (rapid tissue penetration) with a homogeneous distribution, resulting in a lasting effect on the prevention of smooth muscle cell proliferation and migration (21). IVL, which induces small and larger cracks in calcium rich plaques (23), has the potential to facilitate drug transfer to the vascular wall. In this study, only paclitaxel-coated balloons were used. The antirestenotic effect of paclitaxel has been previously shown also in calcified lesions after rotational or orbital atherectomy resulting in similar outcomes as compared to DES (24–26). The efficacy of sirolimus-coated balloons in calcified lesions is currently unknown.

The primary endpoint of this study, 12-month MACE, occurred in five patients (15%) driven mainly by CV death (*n* = 3). Importantly, there was only one ischemia-driven target lesion revascularization within 12 months, suggesting that IVL combined with DCB therapy is a promising novel approach to treat this challenging patient population. One type-2 myocardial infarction occurred during the 12-month follow-up. No acute vessel closures or perioperative myocardial infarctions occurred in this cohort. The safety of DCB-only angioplasty has been previously demonstrated in various patient populations, including observational studies and randomized controlled trials (27–31).

Bleeding following PCI is an important clinical concern due to its association with increased mortality and healthcare costs, especially in elderly population with calcified lesions and with comorbidities such as renal failure (32). This risk for bleeding is particularly high during the first month after PCI. Given the high risk for bleeding (76% were at HBR according to the ARC criteria) among patients in our study, there was a notable rate of bleeding events as clinically significant (BARC 2–5) and severe bleeding (BARC 3–5) events occurred in 24% and 6% of the patients at twelve months, respectively. Most bleeding events were gastrointestinal bleedings and occurred in patients on dual therapy.

PCI using DCB-only approach allows less intensive antiplatelet therapy compared to stenting which is beneficial in patients with HBR (26). The current recommendation on the duration of DAPT after DCB-only PCI is one month in CCS patients and 12 months in ACS patients without HBR (8, 9). The mean duration of double therapy (DAPT or OAC + SAPT) was 3 ± 3 months and 8 ± 5 months in CCS and ACS patients, respectively. After DES implantation, the minimum recommended duration of DAPT is 1 month in HBR patients, and the minimum duration of SAPT with OAC is 6 months (33). In addition to the patient characteristics, the anatomical complexity of the disease (here 79% of the lesions were type B2 or C) and PCI technique affect the operator's decision on the antiplatelet therapy. Our previous observational study suggests that even SAPT is safe after DCB-only angioplasty in HBR patients (34). In this study, 12% of patients at HBR were discharged on either SAPT or OAC alone.

Taken together, the lack of a permanent metal implant may allow a shorter and less potent antithrombotic regimen after PCI in HBR patients. Furthermore, in case of a severe bleeding, the DCB-only PCI allows de-escalation or even pausing all antiplatelets which may not be possible within the first weeks after stenting.

DCBs offer several potential advantages over drug-eluting stents in the treatment of calcified lesions. One key benefit is that DCBs produce more uniform drug delivery to the vessel wall compared to stents. Recent studies have shown that fibrocalcific lesions exhibit more positive remodeling after PCI using paclitaxel DCB compared to lipid-rich plaques (35). This may be attributed to the fact that lipid-rich soft plaques are more prone to flow-limiting and even vessel-threatening dissections after predilation. Furthermore, even after high dilation pressures, stents may remain underexpanded or malapposed in heavily calcified lesions, which can lead to suboptimal outcomes. In contrast, DCB-only PCI overcomes this limitation of DES. Another advantage of DCB-only PCI is that potential restenosis is easier to manage compared to restenosis after stenting because all calcium modification tools, including rotational or orbital atherectomy, can be used without the additional risk due to the previous metallic layers.

## 6 Limitations

There are several limitations in this study. First, as a retrospective, single-arm, single-center analysis, the findings are subject to a selection bias as the patient population was enriched with HBR patients. Moreover, only lesions with an acceptable predilatation result could be treated using DCB-only PCI. Second, the sample size is small, which increases the risk of chance in clinical outcomes. Third, there was no control group (IVL + DES). Fourth, intravascular imaging (IVUS or OCT) was not used routinely and therefore there was not use for quantitative analysis of the severity of calcification. Fourth, there was no systematic angiographic follow-up or follow up to evaluate late lumen loss or gain after DCB-only PCI. Fifth, data regarding the exact number of IVL pulses were not systematically available. Finally, the exploratory nature of the study precludes definitive conclusions regarding treatment effectiveness, and the results should be viewed primarily as generating hypothesis for future research.

## 7 Conclusions

In conclusion, IVL followed by paclitaxel DCB treatment was feasible in the treatment of complex calcified lesions in this study. These preliminary observations generate the hypothesis that this strategy may represent an alternative to DES implantation and support the need for larger prospective observational studies and randomized trials comparing DCB and DES in this setting.

## Data Availability

The raw data supporting the conclusions of this article will be made available by the authors, without undue reservation.

## Supplementary material

Supplementary Figure S1 Pre, post IVL + DCB treatment and follow-up coronary angiographies for the available 10 patients.
