# Supplementary material for: Safety and feasibility of the treatment of calcified de novo coronary artery lesions with drug-coated balloon angioplasty after intravascular lithotripsy
Source: Front Cardiovasc Med. 2026 Jan 30;13:1753826. doi: 10.3389/fcvm.2026.1753826 (PMC12902940; doi:10.3389/fcvm.2026.1753826)
Supplement: Supplementary Figure S1 — Pre, post IVL + DCB treatment and follow-up coronary angiographies for the available 10 patients. [file Datasheet1.pdf]

| | | | |
|---|---|---|---|
| 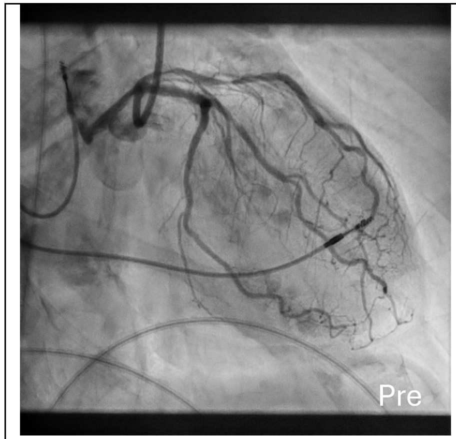  | 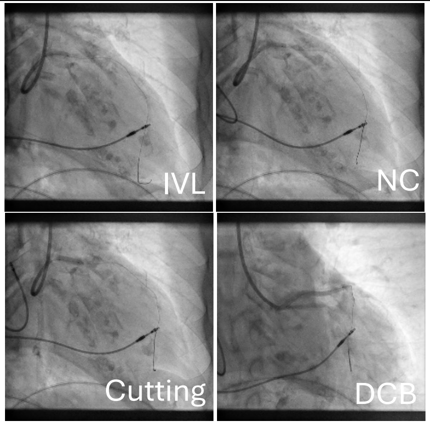  | 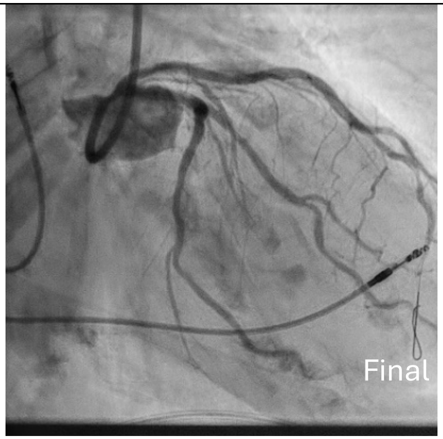  | 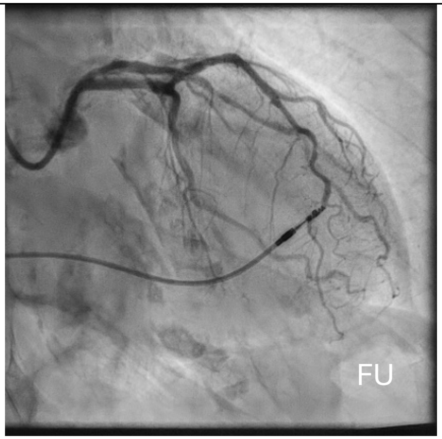  |
| Coronary angiography (CAG) of a 77yr old patient with NSTEMI showed severe coronary stenosis in left anterior descending (LAD) artery with high calcification. | First, small SC balloon was used to predilate the stenosis. After that, predilatation with intravascular lithotripsy (IVL) 3 x 12 mm was performed, only 20 pulses were given before the balloon bursted. Then further predilatation was made with non-compliant (NC) 3 x 15 mm balloon and with 3 x 15 cutting balloon. After good predilatation the lesion was treated with Drug-Coated Balloon (DCB) 3 x 40 mm 12 ATM 30 seconds. | The final result after IVL + DCB treatment in the LAD | In the follow-up the patient had CCS2, and CAG was performed 4 months after index procedure, which showed only minimal restenosis in LAD, FFR in the mid LAD of 0,92, distal LAD was left for medical treatment. PCI was performed to the right posterior lateral (RPL) branch. |

| | | | |
|---|---|---|---|
| 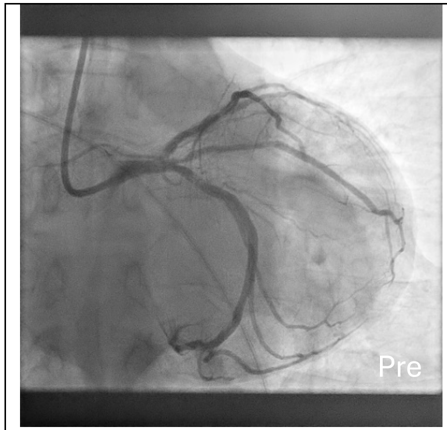 | 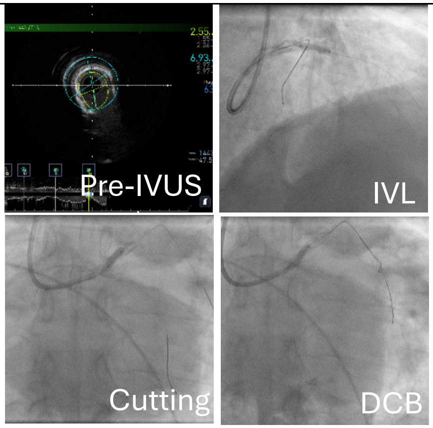 | 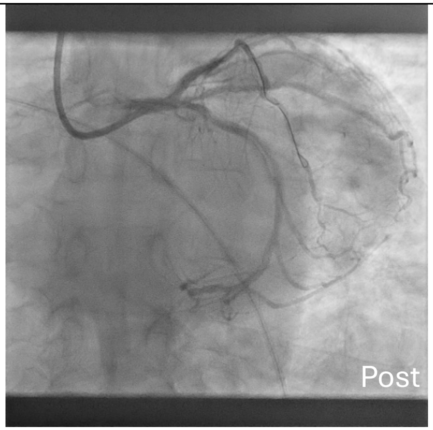 | 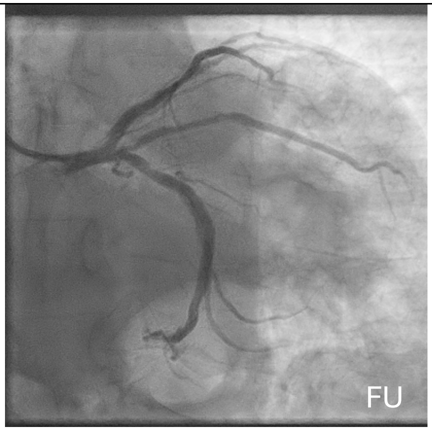 |
| CAG of an 84yr old patient with CCS2 symptoms showed a severe stenosis in the ostial LAD with high calcification and TIMI2 flow. | First, IVUS was performed which showed severe stenosis (MLA of 2,6 mm2) with concentric calcification. IVL using a 3 x 12 mm balloon was performed, almost all shocks were given before the balloon bursted. Then further predilatation was made with 3,5 mm cutting balloon. After predilatation IVUS showed a good predilatation result (MLA 6,3 mm2) and cracs in the calcification. Finally the lesion was treated with two DCBs 3,5 x 15 mm and 3,5 x 40 mm. | The final result after IVL + DCB treatment in the ostial LAD. | CAG was performed 17 months after the index procedure due to dyspnea demonstrating good long term result in LAD. |

| | | | |
|---|---|---|---|
| 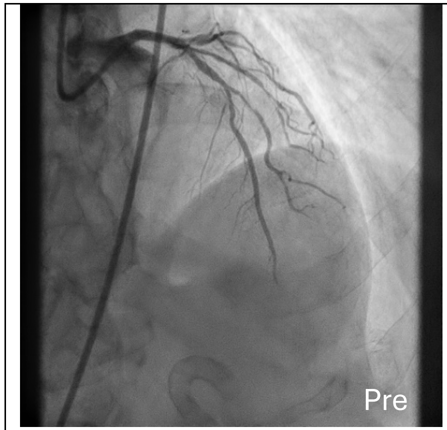  | 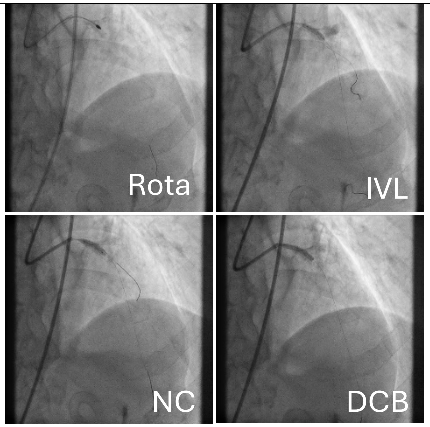  | 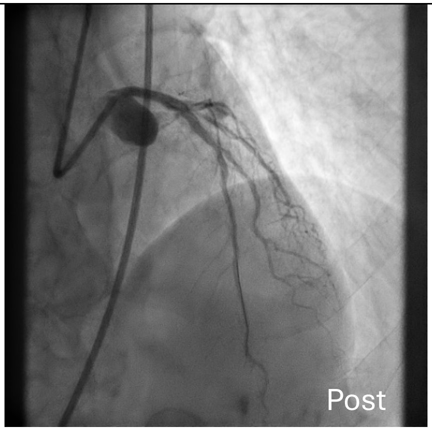  | 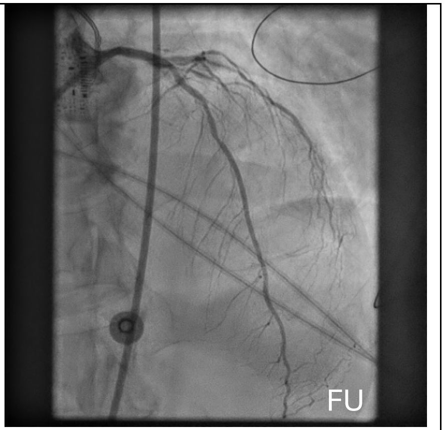  |
| CAG of a 70yr old patient with type 1 diabetes and CCS3 symptoms showed a severe stenosis in the proximal and mid LAD, and in the second diagonal artery with high calcification. | Rotational atherectomy was performed in the LAD (1,75mm burr) and D2 (1,25 mm burr). The distal LAD and D2 were then predilated with NC and treated with DCB. However, there was still significant stenosis in the proximal LAD, and predilatation with IVL 3,5 x 12 mm was performed. Then, further predilatation was made with NC 3,5 mm balloon. After a good predilatation the proximal LAD lesion was treated with DCB 3,5 x 20 mm at 12 ATM for 30 seconds. | The final result after rotational atherectomy and IVL + DCB treatment in LAD and D2. | Patient suffered a sudden cardiac arrest a year after index PCI, so CAG was performed demonstrating a mild restenosis in the proximal LAD. The D1 (no prior PCI) and D2 (no IVL treatment) were occluded. |

| | | | |
|---|---|---|---|
| 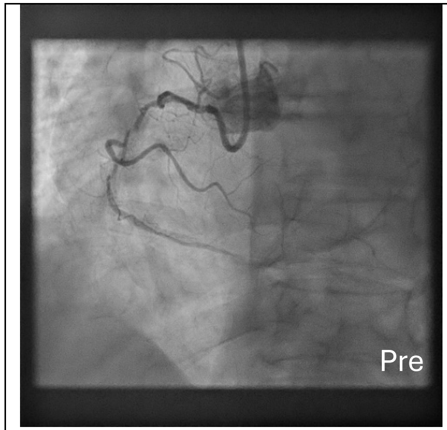 | 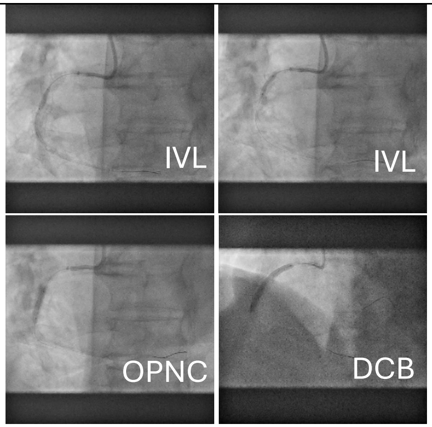 | 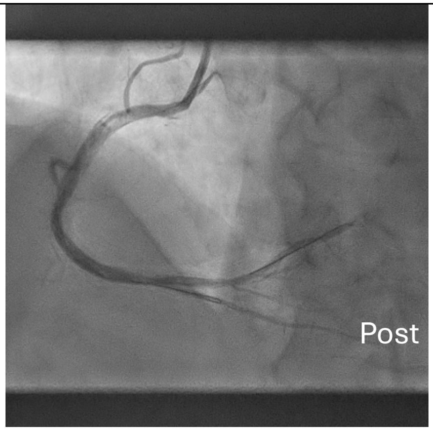 | 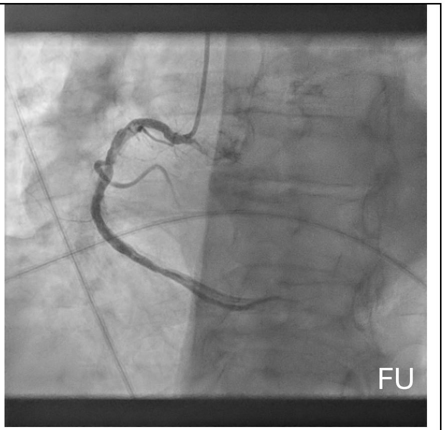 |
| CAG of a 71yr old patient with CCS3 symptoms showed severe three vessel disease, but patient was not eligible for surgery. The patient had also extremely high bleeding risk (thrombocytopenia, liver cirrhosis, varices bleeding). CAG revealed a highly calcific subtotal occlusion of the RCA and significant stenosis in the proximal LAD and LCX. | Predilatation of the RCA lesion was performed with NC balloons, which bursted. Thereafter, predilatation with IVL 3,5 x 12 mm was performed. The first IVL balloon bursted, but predilatation was successfully performed with a second IVL balloon. Then, further predilatation was made with OPNC 3,5 mm balloon. After good predilatation, the whole RCA was treated with multiple DCBs, at IVL segment sizing 3,5 x 30 mm. | The final result after IVL + DCB treatment in the RCA. A non-flow limiting dissection in RCAb was found and stenting was deferred due to high bleeding risk. | Follow-up CAG 3 months after the index procedure showed dissections in the distal and mid RCA indicating an ongoing healing process. FFR in the distal RCA was 0,86. |

| | | | |
|---|---|---|---|
| 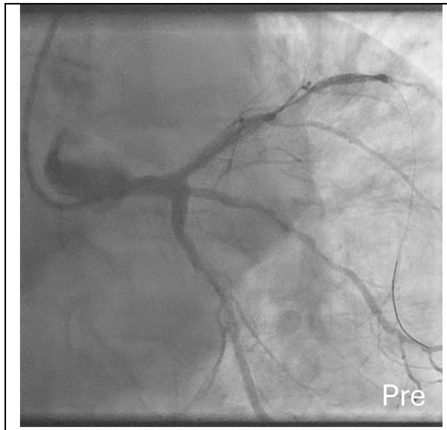  | 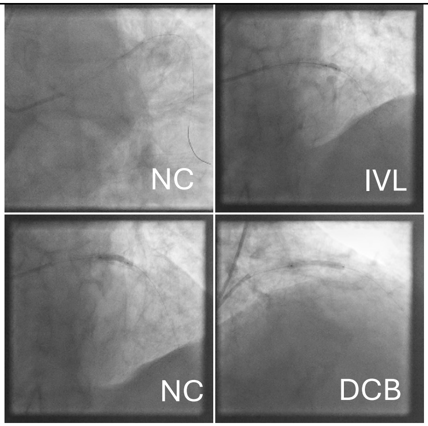  | 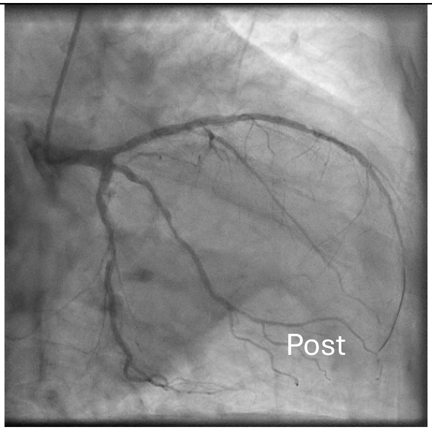  | 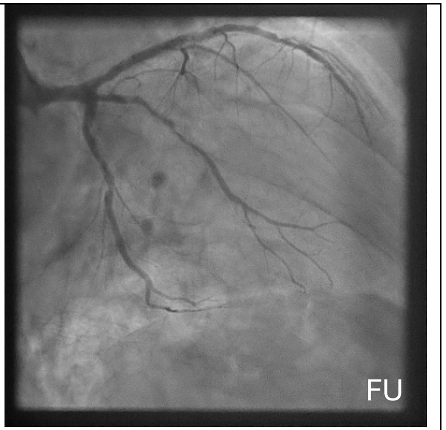  |
| CAG of a 73yr old patient with NSTEMI showed severe coronary stenosis in the mid LAD and moderate stenosis in the proximal LAD with high calcification and TIMI1 flow. | First, NC 3 x 20 mm balloon was used to predilate the stenosis, but the balloon didn’t expand sufficiently. After that, predilatation with IVL 3 x 12 mm was performed successfully to the mid LAD, but the balloon bursted on proximal stenosis. Then, further predilatation was done using a NC 3,5 x 20 mm balloon. After good lesion preparation, the lesion was treated with DCB 3,5 x 25 mm at 8 ATM for 60 seconds. | The final result after IVL + DCB treatment in the proximal and mid LAD. | Due to recurrent CCS4 symptoms CAG was performed 1,5 months after the index procedure. The previously treated LAD showed a good result, but the patient had significant stenosis in the intermediate branch (IM) and in the LCX. |

| | | | |
|---|---|---|---|
| 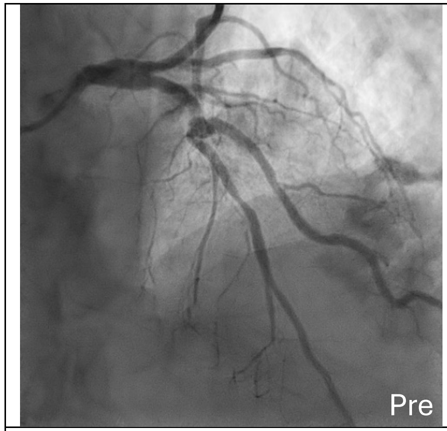 | 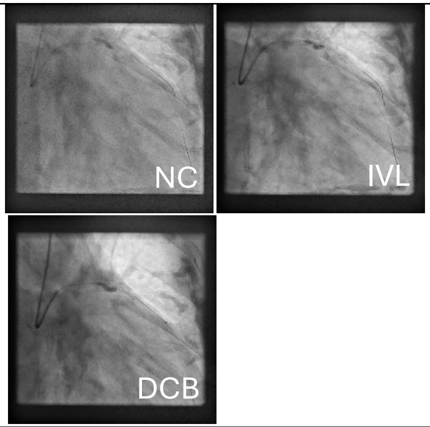 | 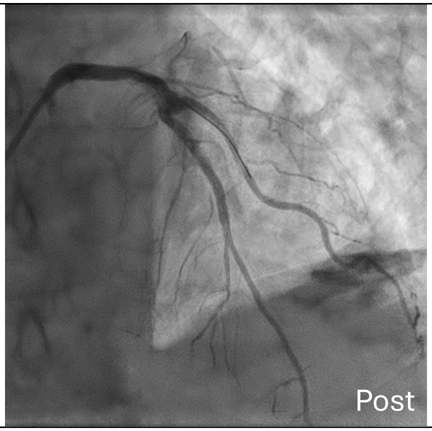 | 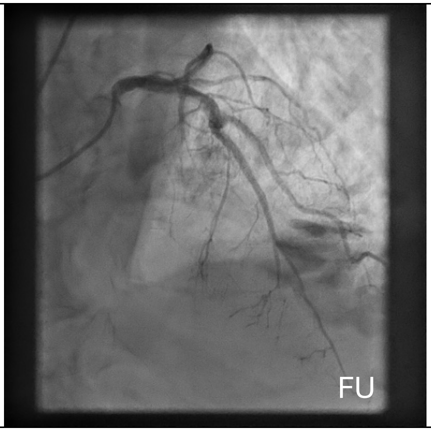 |
| CAG of a 84yr old patient with CCS3 and severe aortic stenosis, showed severe mid LAD stenosis with tortuosity and an aneurysm. | The LAD stenosis was uncrossable for IVUS. First, NC 3mm balloon was used, but the balloon didn't expand. After that, predilatation with IVL 3,5 x 12 mm was performed successfully. After good predilatation the lesion was treated with DCB 3,5 x 15 mm. | The final result after IVL + DCB treatment in the mid LAD. | CAG was performed 1,5 years after index procedure due to NSTEMI. The previously treated LAD showed a good long-term result, but there was a culprit lesion in the RCA. |

| | | | |
|---|---|---|---|
| 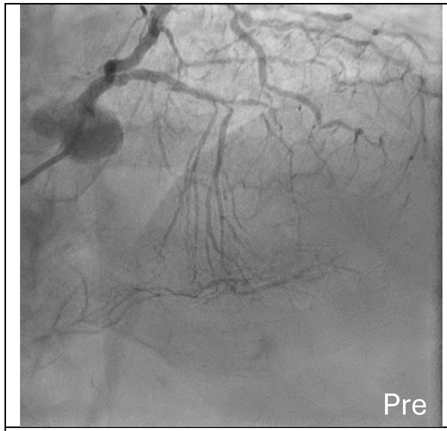  | 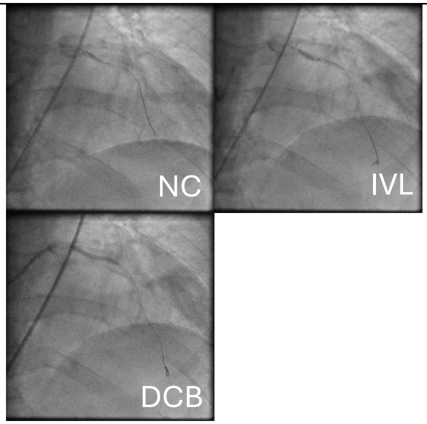  | 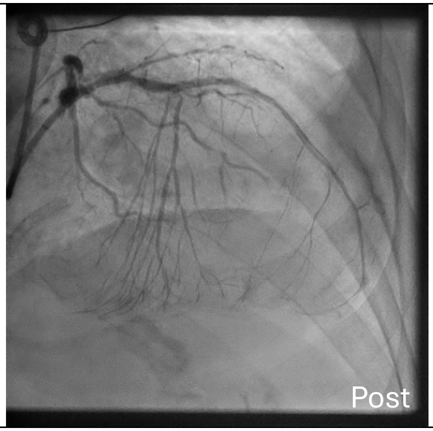  | 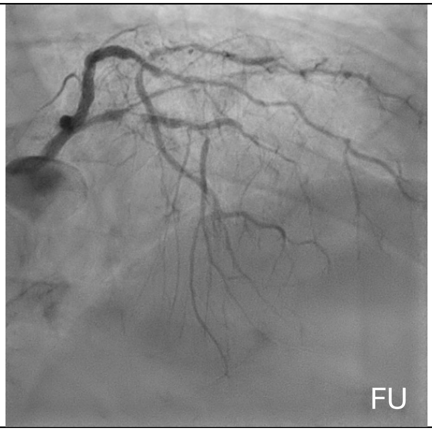  |
| CAG of a 74yr old patient with CCS2 showed severe three vessel disease with a CTO in the mid LAD. The patient was not eligible for CABG. | After successful wiring of the CTO, predilatations were performed with NC balloons, without sufficient expansion proximally, so IVL 2,5 x 12 mm was used resulting a good predilatation result. The LAD was then treated with three DCBs: 3 x 30 mm, 2,5 x 30 mm and 2,5 x 30mm. | The final result of the LAD CTO PCI using IVL + DCB treatment. | CAG was performed 1 year 2 months after index procedure due to NSTEMI. The previously opened LAD was restenosed to CTO. |

<table>
<tr>
<td>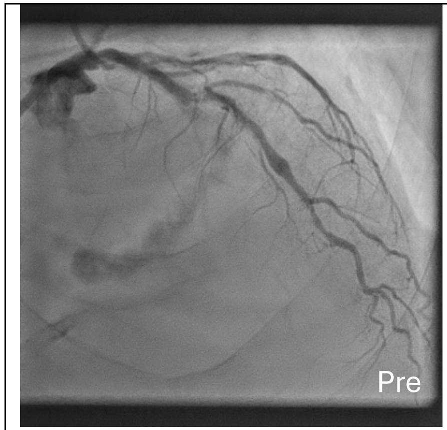</td>
<td>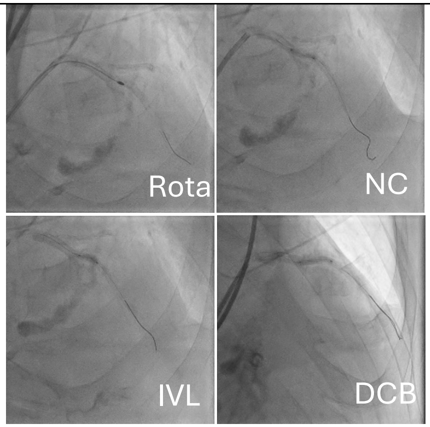</td>
<td>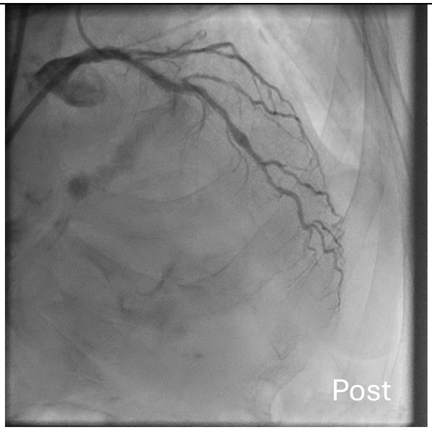</td>
<td>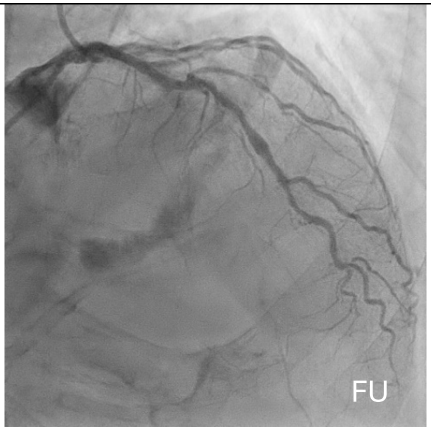</td>
</tr>
<tr>
<td>CAG of a 74yr old patient with CCS2 showed severe three vessel disease with a mid-LAD CTO and severe stenosis in the proximal LAD and D1. Due to lack of viability in the distal LAD area, the PCI of the mid LAD CTO was deferred.</td>
<td>First, rotational atherectomy using 1,5 mm and 2,0 mm burr was performed in the proximal LAD and D1 (only 1,5 mm burr). Then, predilatation was done with a 2,5 mm NC balloon, and 2,75 mm cutting balloon, but the balloons failed to expand. IVL 3 x 12 mm was used and good predilatation was achieved. Finally, proximal LAD and D1 was treated with two DCBs: 3,5 x 10 and 2,75 x 15mm.</td>
<td>The final result after rotational atherectomy and IVL + DCB treatment of the proximal LAD and D1.</td>
<td>Follow-up CAG was performed before a major non-cardiac surgery a year after the index procedure. The treated LAD and D1 showed no restenosis.</td>
</tr>
</table>

| | | | |
|---|---|---|---|
| 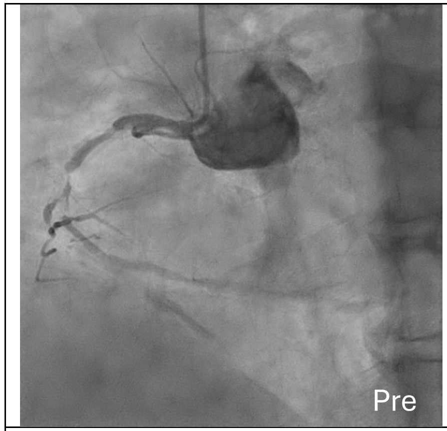 | 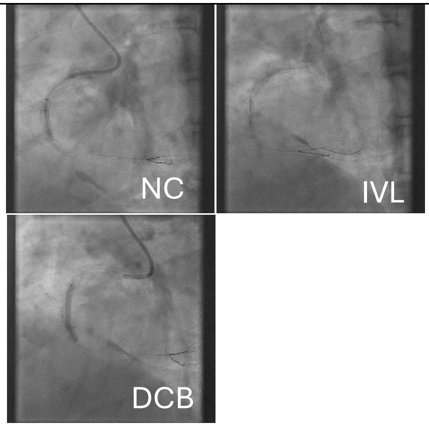 | 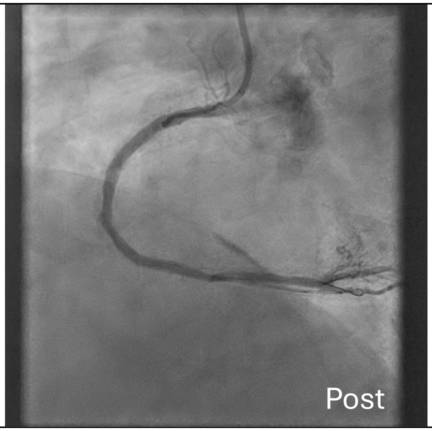 | 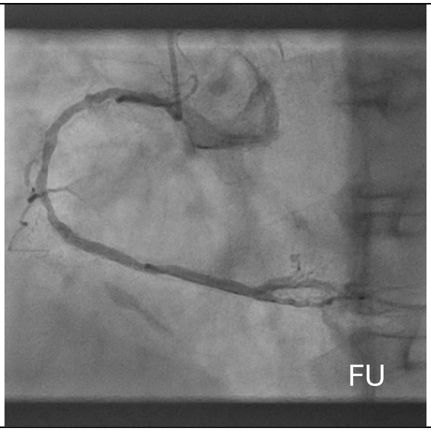 |
| CAG of a 75yr old patient presenting with NSTEMI showed a mid-RCA CTO. | After successful wiring of the CTO, predilatations were performed with NC balloons, but 3,5 mm NC balloon at 24 ATM failed to expand sufficiently. Thereafter, IVL 3,5 x 12 mm was used resulting in good lesion preparation. Finally, the lesion was treated with DCB 3,5 x 30. | The final result after IVL + DCB treatment in the mid RCA CTO. | CAG was performed 2,5 years after the index procedure. There was mild restenosis in the mid RCA, but FFR in the distal RCA was 0,83 showing no ischemia. |

| 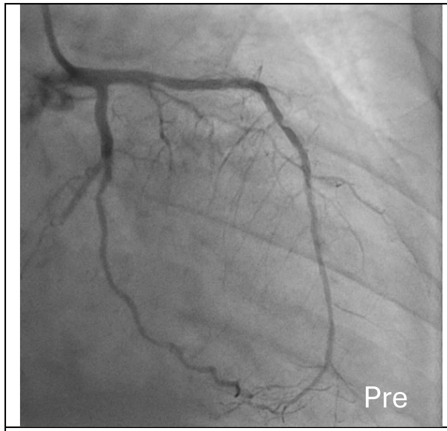  | 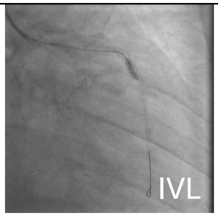  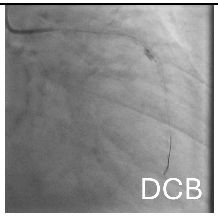  | 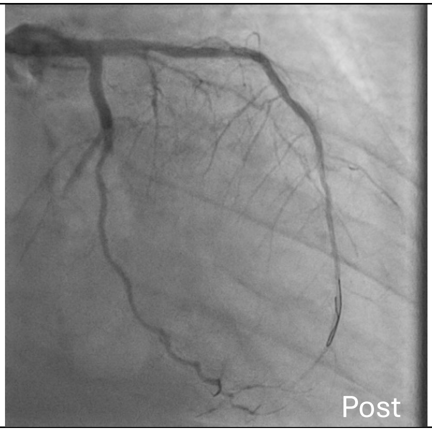  | 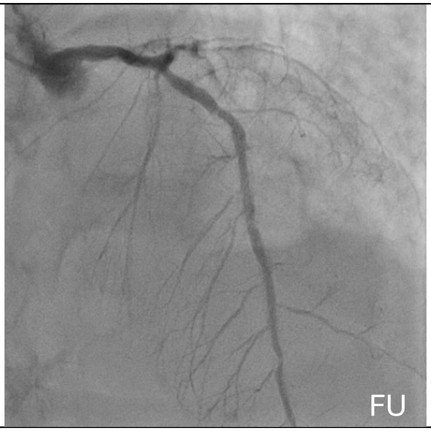  |
|---|---|---|---|
| CAG of a 66yr old patient with type 1 diabetes and chronic kidney failure with dialysis, and CCS4 symptoms showed severe coronary stenosis in the mid LAD with a calcified nodulus. | The predilatation of the mid LAD lesion was performed with a 3,25 x 12 mm IVL balloon. Then, the lesion was treated with 3 x 10 mm DCB. | The final result after IVL + DCB treatment of the mid LAD lesion. | CAG was performed 8 months after index procedure due to NSTEMI. There was moderate restenosis in the mid LAD lesion caused by a calcified nodulus, which was further treated with rotational atherectomy. |
